# Virus Replication Strategies and the Critical CTL Numbers Required for the Control of Infection

**DOI:** 10.1371/journal.pcbi.1002274

**Published:** 2011-11-17

**Authors:** Andrew J. Yates, Minus Van Baalen, Rustom Antia

**Affiliations:** 1 Department of Systems and Computational Biology, Department of Microbiology and Immunology, Albert Einstein College of Medicine, Bronx, New York, United States of America; 2 CNRS, Université Pierre et Marie Curie, Ecole Normale Supérieure, UMR 7625 Ecologie and Evolution, Paris, France; 3 Department of Biology, Emory University, Atlanta, Georgia, United States of America; Imperial College London, United Kingdom

## Abstract

Vaccines that elicit protective cytotoxic T lymphocytes (CTL) may improve on or augment those designed primarily to elicit antibody responses. However, we have little basis for estimating the numbers of CTL required for sterilising immunity at an infection site. To address this we begin with a theoretical estimate obtained from measurements of CTL surveillance rates and the growth rate of a virus. We show how this estimate needs to be modified to account for (i) the dynamics of CTL-infected cell conjugates, and (ii) features of the virus lifecycle in infected cells. We show that provided the inoculum size of the virus is low, the dynamics of CTL-infected cell conjugates can be ignored, but knowledge of virus life-histories is required for estimating critical thresholds of CTL densities. We show that accounting for virus replication strategies increases estimates of the minimum density of CTL required for immunity over those obtained with the canonical model of virus dynamics, and demonstrate that this modeling framework allows us to predict and compare the ability of CTL to control viruses with different life history strategies. As an example we predict that lytic viruses are more difficult to control than budding viruses when net reproduction rates and infected cell lifetimes are controlled for. Further, we use data from acute SIV infection in rhesus macaques to calculate a lower bound on the density of CTL that a vaccine must generate to control infection at the entry site. We propose that critical CTL densities can be better estimated either using quantitative models incorporating virus life histories or with *in vivo* assays using virus-infected cells rather than peptide-pulsed targets.

## Author Summary

In the search for vaccines that provide reliable protection against major diseases such as HIV-AIDS, TB and Malaria, there is now a focus on generating populations of antigen-specific cytotoxic T lymphocytes (CTL), immune cells that recognise and kill infected cells. However, we have little idea of the number or density of CTL a vaccine would need to elicit to provide sterilizing immunity to an infection in a given tissue. In this study we use mathematical models to understand how a virus's replication strategy influences the minimum density of CTL needed to provide immunity at an infection site. We show that traditional models that neglect the viral lifecycle within infected cells will underestimate this density. To illustrate, we use our modelling framework to estimate the CTL density needed to control the spread of virus at the very earliest stages of primary SIV infection in rhesus macaques.

## Introduction

The majority of vaccine design approaches to date have used neutralizing antibody titers as a correlate of efficacy. However, major infectious diseases such as HIV-AIDS, TB and Malaria have not yet fully yielded to vaccines aimed at eliciting antibodies. There is currently much interest in developing vaccines that also elicit pathogen-specific CD4 T cells or, more commonly, CD8 T cells (also known as cytotoxic T lymphocyte, or CTL). Such vaccines need to generate T cells of sufficient functional quality, appropriate tissue tropism, and in sufficient numbers. Manipulating all three features of the CTL response presents a major challenge that requires understanding of the biology of T cell priming and the cells' interactions with their microenvironment during clonal expansion and contraction. However, assuming the first two features can be optimised, the third raises an important question – how many T cells does a vaccine need to generate in order to protect against infection? This of course might be determined empirically in animal models, but another approach is to search for principles that might guide our intuition for human vaccine design.

A CTL response is a dynamic process whose chance of success may depend on precursor frequency, speed of priming and clonal expansion or reactivation, total cell numbers, access to infected tissues, and the rate and efficiency with which they survey potentially infected cells. Mathematical models can help us develop a quantitative understanding of how these processes influence the potential for protection. In this paper we focus on tissue-resident activated CTL and the challenges they face in eliminating a growing population of virus-infected cells, with an emphasis on how virus replication strategies influence the efficiency of CTL surveillance.

## Results

### The standard model predicts critical thresholds for CTL immunity

What we present here builds on the standard model of virus growth used extensively in the literature (see, for example, refs [1]–[9]). In the standard model the dynamics of infection in a tissue can be described by the abundance of infected cells 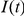. During early stages of an infection when susceptible cells are in abundance, and in the absence of specific immunity, 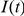 grows exponentially as 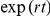 (the doubling time 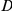 is then 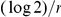). The parameter 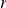 is the *per capita* growth rate of the infected cell population and is the net outcome of a plethora of biological processes; virus replication, shedding from infected cells, clearance of virus from the intercellular space, infection of susceptible cells; and loss of infected cells by natural mortality, virus-induced mortality and innate immune mechanisms. Now suppose in addition that infected cells can be killed by CTL. The standard model assumes CTL are tissue-resident and activated, CTL and infected cells are well-mixed, distributed spatially randomly, and a CTL encounters other cells with a constant probability per unit time. These assumptions yield a mass-action term for the killing of infected cells by CTL,
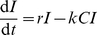
(1)where 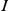 and 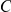 are the densities of infected cells and CTL respectively. We define density to be a dimensionless quantity in the range 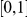. It represents cell numbers locally as a fraction of all of the cells being surveyed by CTL, uninfected or infected. We use this definition (rather than cells per unit volume of tissue, cells per unit volume of blood, or the proportion of CTL that are antigen-specific) because it relates directly to cell frequencies obtained from tissue samples by flow cytometry, and it yields a simple interpretation of the parameter 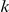, which we return to in a moment.

In this model, sterilising immunity corresponds to a net growth rate 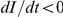. To achieve this requires specific CTL to be present above a critical density 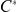 where
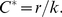
(2)With our dimensionless definition of density, the parameter 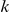 is the rate of surveillance, or the expected number of cells of any kind that a CTL encounters per unit time; 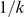 is the expected time between contacts; and 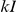, where 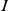 is the range 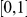, is the expected number of infected cells that a single CTL encounters per unit time (in Text S1 we discuss how this interpretation of 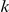 relates to its interpretation when 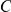 and 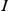 are measured as numbers of cells per unit volume). So if in the absence of CTL, infected cells double in number every 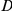 days, a simple estimate of the local density of CTL required to control the infection is
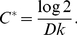
(3)However, this simple model neglects at least two major biological processes that may impact the estimate of the critical density – the dynamics of CTL-infected cell conjugates, and the life history of viruses within infected cells. We investigate these separately, and begin with the influence of CTL-target handling time on the rate at which a population of CTL kills a fixed number of target cells.

### The dynamics of CTL-infected cell conjugates

The mass-action killing term implies that killing is instantaneous on encounter of a CTL with an infected cell. However, CTL take time to lyse and detach from their targets. Mempel *et al.* [10] showed that CTL engaged in lysis can remain bound for up to half an hour after initial contact. Thus accounting for the temporary sequestration of CTL in conjugation with infected cells may be important for the dynamics of killing and for assessing the validity of the mass-action assumption.

A simple way to include the time take for CTL to handle infected cells is to explicitly track CTL-infected cell conjugates [11], such that free CTL (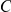) initially present at density 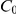 successfully bind to infected cells present at density 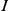 at rate 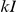 per CTL, and remain in conjugates 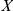 for a mean time 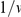. This time can be a compound of several distinct processes – delivery of the lethal hit following conjugation, release of the apoptotic cell, and a possible recovery time before the CTL is capable of detecting another infected cell. Here 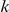 is the rate of surveillance of all cell types, and incorporates any time spent conjugated with uninfected cells.
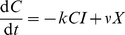
(4)

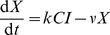
(5)

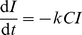
(6)Since if we assume no loss of CTL the number of bound complexes 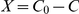, 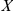 can be eliminated to give
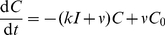
(7)

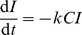
(8)We can now identify the conditions under which mass-action holds.

*If CTL are in excess* (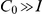), then 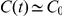 and so mass-action holds for all 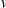 and 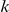, irrespective of handling or search times.

*If infected cells are in excess*, three regimes exist:

1. When search times are small compared to handling times, or 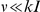, 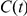 approaches the following at exponential rate 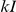:
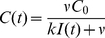
(9)which yields
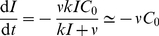
(10)In this regime, the rate of cell loss is limited by the handling time 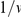 and the CTL frequency 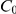, and the infected cell density declines linearly with time in the absence of replication. If susceptible cells do not become limiting, in this regime infected cells reach a non-zero steady state and clearance is not possible.
2. When handling and search times are comparable (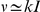), an analytic solution is not possible, but we can describe the dynamics of clearance. At 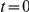, all CTL are unbound and so the rate of loss of infected cells is maximal, with per capita rate 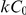. CTL are then progressively sequestered in conjugates; the killing rate falls, to a minimum 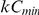 at 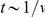 where 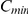 is the solution to 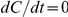 and 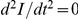, which yields
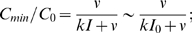
(11)so if 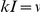, the killing rate is halved at 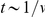; then for 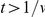 , as infected cells become scarce, the number of free CTL progressively returns to 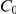 and we approach mass-action killing using total CTL density 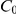 (that is, 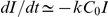) for 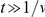.
3. When handling times are short compared with search times (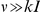), then
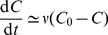
(12)which rapidly equilibrates at 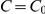 and the loss of infected cells again obeys mass-action, 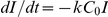.

Considering the dynamics of surveillance and of CTL-target conjugates may resolve some of the discord between microscopy studies showing extended encounter times between CTL and their targets [10] and simple mass-action models of *in vivo* surveillance and killing by CTL, all of which describe experimental data well using the assumption of instantaneous killing and yield estimates of 1–40 cells surveyed per CTL per minute [8], [9], [12], [13]. Extended encounter times between CTL and infected cells are consistent with these models if CTL are in excess. At high ratios of CTL to infected cells (usually referred to in the literature as the effector∶target ratio, E∶T), few CTL will kill more than once, and so handling time becomes irrelevant for the long-term dynamics of infected cell removal.

So if the inoculum size of the virus is low compared to existing effector cell densities (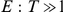), or if handling times are short compared to the time taken to survey cells, we do not need to consider the dynamics of CTL-infected cell conjugates to calculate the minimum density of specific CTL needed for protection.

### The influence of virus life history strategies on killing dynamics

CTL are triggered by their recognition of peptides derived from virus proteins generated within the infected cell and presented on MHC class I molecules. Existing estimates of the surveillance rate 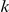 [8], [9], [12], [13] use the assumption that the infected cells are expressing peptide-MHC complexes at a constant level over the timecourse of the experiments. However, viral replication is a dynamic process and so the window of expression of a given peptide-MHC complex by a cell infected with a live virus may be limited. Thus CTL have to contend with not only locating cells within a tissue, but also the dynamics of the expression of viral epitopes [14].

In the model that follows we assume we are in a regime where mass-action operates. In Text S2 we describe the more general model in which both handling time and virus epitope dynamics are accounted for.

How will the dynamics of virion production and virus epitope expression affect our estimate of the local density of CTL required for sterilising immunity, 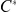
? With the mass-action assumption, the population dynamics of infected cells 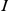 becomes
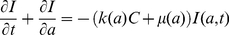
(13)

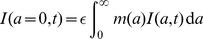
(14)where 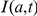 is the density of cells at time 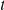 that have been infected for a time 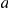, 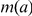 is the rate of release of virions at age 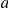 after infection, 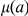 is the age-dependent mortality of infected cells not ascribed to CTL activity, and 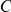 is the (now assumed time-independent) free CTL density. This equation describes the dynamics of infected cell numbers in infinitesimal age classes as they are generated through new infection, age and die. Since detection upon encounter is dependent on the expression of the appropriate virus epitopes and hence on time since infection, the rate at which a CTL encounters and recognises infected cells in age class 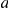 is now 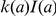, and 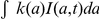 is the total rate at which a CTL encounters and recognises infected cells. Equation (14) represents the process of new infections, and assumes (i) susceptible cells are in abundance, and (ii) that infection of new cells or clearance of free virus is more rapid than the dynamics of virus replication and release from infected cells. So the rate of infection of susceptible cells at time 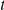 is proportional to the total rate of virus production at that time, with constant of proportionality 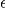.

A useful quantity is the survivorship 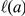, the proportion of a cohort of cells that are still alive at a time 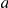 after they were infected. The survivorship is related to the total mortality 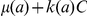 through
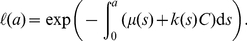
(15)


The steady state age-distribution solution of equation (14) yields the total cell population growing or declining as 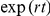, where 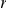 satisfies the Lotka-Euler equation (see Text S3 for a derivation)
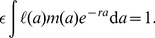
(16)This has a unique real solution for the growth rate 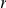 [15].

We consider representations of two replication strategies – a budding virus, which after some delay following infection sheds virions from the host cell at a constant rate, with a possible increased burden of mortality for the host cell; and a lytic virus, which replicates in the host cell without release of virions until it lyses the host cell, releasing all its progeny within a short time interval.

**Figure 1:**
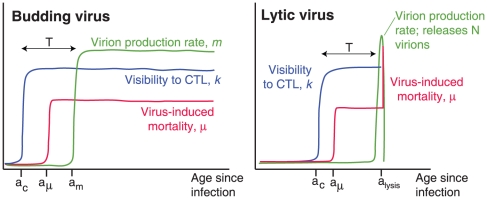
Schematic representation of budding and lytic virus replication strategies. On the left, **budding viruses**: a time 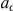 after infection of the cell, sufficient epitopes are presented on the cell surface for CTL to recognise and kill the cell; virion production causes an increase in cell mortality, 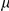, at a later time 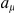; and at a later time 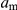, virions begin to be shed from the cell at constant rate 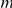. On the right, **lytic viruses**: beginning a time 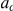 after infection the cell becomes visible to CTL, after time 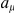, stress induced by virus replication within the cell generates an additional mortality rate 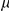; and the infected cell bursts and releases 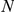 virions a time 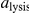 after infection. In both figures, 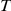 is the duration of a cell's visibility to CTL before virus release begins. Intervals between events are not shown to any scale.

For both strategies we need to know three functions. These describe the visibility to CTL, the virus production rate and the virus-induced mortality as functions of age since infection, and are shown schematically in Figure 1.

In our representations of these strategies we have made the simplifying assumption that above a certain threshold of epitope expression, an infected cell is capable of being identified by CTL at constant rate 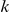. A biological motivation for this is that CTL can recognise as few as 10 pMHC complexes [16] and so we expect the efficiency of recognition to saturate quickly in peptide density. With the forms for the virus-induced mortality described in Figure 1, and 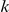, exact expressions can be derived for the survivorship 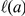. For budding viruses, we assume that infected cells become visible to CTL before the onset of virus-induced mortality 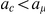 (Figure 1), but our conclusions are independent of the ordering of these events. We then have
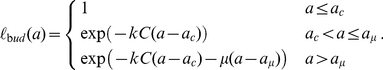
(17)


For lytic viruses,
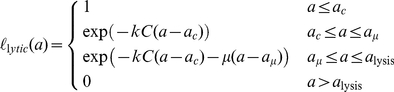
(18)For the budding virus, solving equation 16 for the steady state growth rate 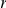 reduces to solving the following implicit equation for 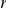,
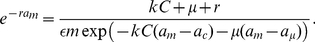
(19)


For lytic viruses it yields the direct solution
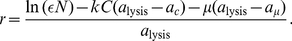
(20)


Identical expressions are derived if visibility to CTL begins after the onset of virus-induced mortality 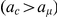.

#### Comparing CTL efficacy against different virus strategies

We can compare the effectiveness of CTL responses against lytic and budding virus infections. For reference we compare both to the standard model of a budding virus that assumes infected cells have exponentially distributed lifetimes, and immediately following infection become visible to CTL and begin to make virus at a constant rate.

For a meaningful comparison of the three model strategies, we choose parameters such that (i) in the absence of CTL, infected cells have the same expected lifetime and the same net growth rate 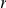, and (ii) the duration of the window of opportunity for CTL to detect infected cells before virus is released is equal in the age-structured budding and lytic models. This is the parameter 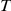 shown in Figure 1.

**Figure 2:**
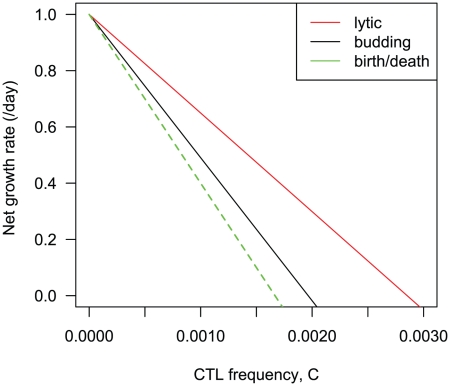
Dependence of infected cell growth rates on CTL numbers, for different virus replication strategies. We compare the standard model (green) with models of a budding virus (black) and lytic (red) strategies. Parameters are chosen so that in the absence of CTL all models yield the same infected cell growth rate, expected lifetime, and for the lytic and budding strategies have the same window of visibility of infected cells to CTL, 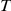, before virus release begins. Parameter choices; growth rate in absence of CTL is 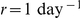, equivalent to a doubling time of 16 hours; expected infected cell lifetime is 2 days; 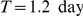; onset of virus shedding in the budding virus model is at 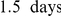; death rate due to cytopathicity of lytic virus, 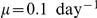; 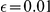 (new infected cells per virion).

In the absence of CTL, the expected lifetime of a cell infected with a budding virus is 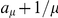. For a lytic virus it is 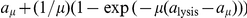. In a simple birth-death model 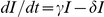, where new infected cells are generated at rate 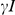, the expected lifetime of infected cells is 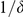 and their numbers grow at net rate 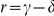. We set the growth rate without loss of generality to 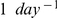, and then choose parameters that simultaneously equate the infected cell lifetimes, satisfy equations (19) and (20), and equate the values of 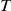 for the budding (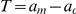) and lytic 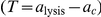 strategies. Figure 2 shows the dependence of the net growth rate of infected cells on the CTL density 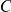 for the different strategies.

We draw three conclusions here. First, if budding and lytic viruses are visible to CTL from the time of infection, both life-history strategies give identical results to the simple birth-death model. If there is any delay in infected cells becoming visible to CTL, the threshold CTL frequency required for immunity increases.

Second, if we control for growth rate, infected cell lifetime and the CTL window of opportunity 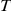, a lytic strategy is harder to control than a budding strategy. This is perhaps counterintuitive at first; one might expect that removal of a cell infected with a lytic virus removes all the potentially infective virions and thus would have a more significant impact on slowing the spread of infection than the removal of a continuously-shedding infected cell. However, the result can be understood simply; with these constraints, the cell infected with a lytic virus is visible to CTL for a smaller proportion of its replicative cycle. With equal expected lifetimes and growth rates, the average rate of production of virus per unit time is equal for the two strategies – so on average a CTL killing a lytic-virus-infected cell prevents fewer virions being released than when killing a cell infected with a budding virus.

Our third conclusion is that to make a parameter-independent comparison meaningful requires controlling for the growth rate, cell lifetime and CTL visibility window 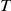, as well assuming equal 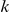 for the two strategies. Relaxing these constraints and allowing the parameters to vary then moves from the general to the specific, and for a given pair of virus life-histories the lytic versus budding conclusion above may be reversed. Given the parameters governing different virus replication strategies, this framework allows us to predict and compare the abilities of CTL to control them.

### Example: What CTL density is needed to control acute SIV infection?

SIV in rhesus macaques buds from its primary target cell population, CD4 T cells. Infection may begin at a mucosal surface and virus remains localised there for 2–7 days before disseminating to other tissues [17], [18], followed by rapid exponential growth of virus titers in blood. Eventually a combination of mechanisms (CTL removal of infected cells, virus cytopathicity, innate immunity, the availability of susceptible cells, and antibody responses) brings virus growth under control and the infection enters the chronic phase. Here we use data from acute SIV infection to estimate the minimum density of tissue-resident CTL that a vaccine needs to elicit for early control of infected cell growth at the site of infection. We assume that the inoculum size is small, and so E∶T will be large. In this regime we argue that handling time can be neglected, and that the mass-action approximation is valid. We assess the validity of the E∶T assumption following the analysis below.

#### Estimating the critical killing rate 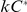


We assume that the cytopathic effects of SIV begin upon virus shedding (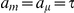). The growth rate 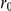 of the infected cell population when susceptible cells are in abundance is given implicitly by equation 19,
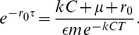
(21)


In what follows, we assume that both the natural mortality of cells and the contribution of the endogenous CTL response to infected cell death in the first few days of acute infection are negligible. The combined process of presentation of SIV epitopes to naive CTL in local lymph nodes, activation, proliferation and migration of CTL to the infection site is likely to take several days; and similar upslopes of virus load are observed in SIV-infected rhesus macaques in the presence or absence of CD8 responses [19], although CTL likely make a substantial contribution to limiting peak viremia [19]–[22]. We therefore assume that cell death at the site of virus entry in the first week of infection is due mainly to non-CTL mechanisms such as innate immunity and viral cytopathicity, and so we assume 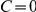 in unvaccinated individuals.

Li *et al.* [23] studied the growth of infected cell numbers and CD4 depletion in the gut lamina propria in SIV infection and showed that local infected cell densities doubled between days 6 and 7 post-inoculation (from 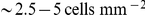), corresponding to a net exponential growth rate of 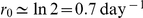. The death rate of infected CD4+ T cells could be estimated following depletion of uninfected cells locally, at around 28 days post-infection, and found to be approximately 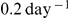 (half-life 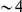 days). A caveat here is that at this relatively late stage of acute infection this figure very likely includes the contribution of adaptive immune responses to cell death. So it likely overestimates the death rate of infected cells in the first week of infection.

Now suppose that a vaccine can generate a local SIV-specific memory CTL density, 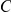. What is the critical value 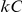 needed to reduce the acute growth rate to zero? Using equation (21) with 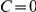 and the parameter estimates from primary SIV infection above, we can eliminate the unknown quantity 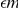 and obtain
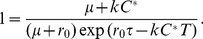
(22)We assume shedding begins 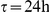 post infection [24] and that an infected cell becomes visible to CTL 6–12 hours post infection, depending on the epitope being expressed. We solve (22) numerically to calculate a distribution of estimates of 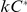, using flat priors on the uncontrolled growth rate 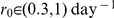, infected cell death rate 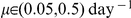, and the CTL window duration 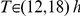. We obtain the median value 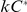 = 0.8 day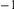 (IQR 0.6–1.1). Thus the CTL elicited by a vaccine must increase the infected cell death rate at least 2-fold (IQR 1.0–4.1).

We can assess the importance of modeling virus epitope dynamics by comparing these estimates of 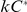 to those of the standard model in which 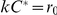. To control virus growth with a CTL response directed against an epitope first visible 6 h post infection (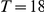 h) requires an increase in the critical CTL density of 14% (IQR 12–15) over the standard model prediction; a response directed against an epitope visible at 12 h requires an increase of 33% [28]–[38].

The quantity 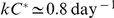 is the minimum killing rate required of one epitope-specific response to control infection. However, a vaccine-induced CTL response may target multiple epitopes. Each specific response will be able to recognise an infected cell beginning a time 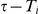 after it becomes infected, and will then contribute a killing rate 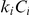. Each may then be required to contribute less than 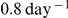 to the infected cell death rate.

#### Timing of the CTL response

Our estimate of the critical killing rate does not depend on the time required for reactivation and/or migration of tissue resident of SIV-specific CTL to the site of infection. It is also independent of inoculum size, provided 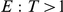 as required by the assumptions of the model. This independence arises because all rates of growth and death are linear in infected cell numbers. In fact, a delay in the onset of CTL activity allows the growing population of infected cells to approach the steady-state age distribution required for our result to hold. Nevertheless, an early-acting CTL response must act rapidly enough to avert the systemic spread of the virus; thus unsurprisingly it is important to induce a response that is as large and fast-acting as possible.

#### CTL efficiency early and late in acute infection

To put the estimate of 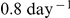 into perspective, we can compare it to estimates of the total contribution of CTL to infected cell death late in the acute phase of SIV infection. Ganusov and de Boer [25] calculated bounds of 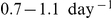 for killing by CTL specific for the KP9 epitope of Gag near the peak of acute infection of rhesus macques with SIV. Mandl et al. [26] obtained a comparable estimate of 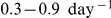 for the response to Tat epitopes. Since there are likely multiple epitopes being targeted, these figures are lower bounds on the total CTL-mediated killing rate. This rate is then much larger than our estimate of the total CTL killing rate required at the entry site.

These observations suggest that the challenge CTL face in controlling the spread of virus after systemic dissemination is far more severe than the one they face at the infection site. There are several possible reasons for this. First, infected cells are spatially localised. Second, resting CD4 T cells are likely the primary target cell population early in infection, and these cells produce virus at a substantially lower rate than the activated CD4 T cells that are the major source of virus in the disseminated acute phase [27]. Third, progressive exhaustion of CTL likely reduces per-CTL surveillance or killing rates later in infection.

#### Estimating the critical CTL density

As a step further, knowing the required vaccine-induced cell death rate 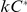 immediately gives us a lower bound on 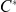 if we know the surveillance rate 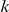 – the rate that a single CTL moves between potential targets – at the entry site. The value of 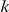 is currently unknown, although we can place broad bounds on it using estimates from other *in vivo* model systems. Direct estimates have been derived from killing of non-replicating cells pulsed with LCMV-derived peptides, in mouse spleens [8], [9], yielding 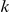 in the range 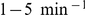 per CTL; again for LMCV in mice using longitudinal blood measurements [12], giving a higher estimates of 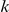 in the spleen in the range 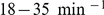 for acute infection, and 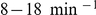 in chronic infection; and for polyoma virus [13] using peptide-pulsed splenocytes, 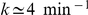 in acute infection and 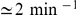 in chronic infection. (Notably, these studies consistently conclude that the per-cell rate of surveillance and killing of CTL is reduced in chronic infection). Using bounds of 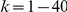 cells/minute, 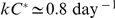 yields critical CTL densities 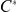 in the range 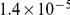 to 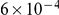. Despite this uncertainty, which arises from our deliberately conservative bounds on 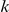, these results suggest the hopeful message that the required CTL densities are not excessively large. Polyoma virus [13], Influenza (Seddon, B., unpublished observations) and LCMV can all induce memory CTL densities among total splenocytes of at least 1%, and with the obvious caveat of differing anatomical locations, this density is at least two orders of magnitude larger than the predicted minimum densities of mucosa-resident SIV-specific memory CTL required for protection.

#### E∶T ratios in early SIV infection

We have shown that we expect mass-action kinetics to hold if populations are well-mixed, and either the E∶T ratio is high or handling times are much shorter than cell-cell surveillance times. In early SIV infection, the validity of the well mixed and/or the high E∶T assumptions will depend jointly on the degrees to which infected cells and CTL are clustered or spread diffusely across the tissue. If both populations are well mixed, we can make a rough estimate of E∶T early in SIV infection if CTL are at the predicted critical density. We estimate 3000–10000 cells (of all types) per 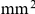, assuming close packing with a center-to-center spacing 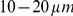. CTL at a density of 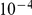 will then be present at 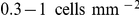, so 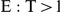 if the density of infected cells is smaller than this. Li *et al.* [23] measure the density of SIV RNA+ CD4 T cells at day 6 post infection to be approximately 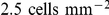 with a doubling time of approximately 1 day. Using the well-mixed assumption, we would then expect 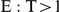, and so the model to be valid, until at least day 3 or 4 post infection.

We also note that the limits of applicability of mass-action models to killing assays are still ill-defined. Ganusov *et al.* [28] demonstrated that for killing of LCMV-pulsed cells in the mouse spleen, a mass-action model appears to hold for E∶T ratios as low as 0.1.

#### Target cell availability

If infected cells are tightly clustered and sparsely infiltrated by CTL, one would expect the rate of killing by CTL to be limited by the handling time once a cluster has been located. In the well-mixed deterministic model, the total rate of loss of infected cells will then be linear in CTL densities and independent of infected cell numbers (equation (10)). In this regime, the model predicts CTL will ultimately fail to control the infection, assuming susceptible cells are abundant and accessible. These assumptions may not hold, however. The density of susceptible cells in healthy tissue is an upper limit to infected cell densities in very early infection, before the influx of SIV-specific CD4 cells that provide new targets. Resting CD4 T cells are present at a density of 100–200 mm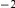 in the vaginal mucosa (using immunohistochemical staining of biopsies taken from healthy rhesus macaques – Gordon S., Franchini G. (NCI), unpublished data), meaning susceptible cells make up 1–5% of the total cell population in the lamina propria. Li *et al.* [23] measure mean peak infected cell densities of 60 cells 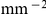; so if infected cells are clustered in patches, the local availability of susceptible cells may be a factor affecting the rate of virus spread.

All of these uncertainties emphasise that more precise estimates for 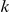 and 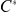 need to be obtained with assays in tissues whose spatial organisation more closely reflects typical HIV/SIV infection sites in oral, rectal or vaginal mucosa, and using cells infected with live replicating virus rather than peptide-pulsed targets.

#### Other outstanding problems

There are inevitably many qualifications to this result that are specific to SIV, as well as more general issues that we present in the Discussion. Several will tend to increase the estimate of the critical frequency. First, as noted above, our estimate of the infected cell death rate may be too high, as it is taken from data at the infection site 28 days-post infection when the primary CTL and antibody responses are likely present. We incorporate our uncertainty in this death rate by using a wide spread of plausible values. Second, in the early growth phase of natural primary infection, selective pressure on the mutating virus exerted by the developing CTL response is expected to be low. A vaccine-induced memory CTL population will increase this pressure. To minimise this effect, broad coverage of virus epitopes to both early and conserved proteins is required. Third, we neglect the longer-term effect of the early generation of latently infected CD4 T cells that escape CTL detection, for which there is some evidence in acute infection [29].

Other factors will act to reduce our estimate. For example, in addition to cytolysis, CTL secrete soluble factors that may make a substantial contribution to CTL-mediated protection in both the acute [30] and chronic [31], [32] phases. Our model includes cytolytic effects only and so may overestimate the number of memory CTL required to control infection. In addition, a vaccine is likely to induce specific antibody that will further decrease the rate of transmission of virus from cell to cell, lower the growth rate of infected cell numbers and so lower the estimate of CTL frequencies necessary to reduce growth to zero.

Finally, it is worth noting the difficulties in connecting rates of surveillance and killing by a single CTL in a given tissue to estimates of the total-body contribution of CTL to infected cell death. The problems stem from the dimensionality of rate constants and the implicit averaging of the effects of CTL in different anatomical locations. For example, Wick *et al* [33] estimated the rate at which individual CTL kill using blood from HIV-infected individuals. In their terminology, 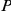 and 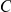 are the densities of infected cells and specific CTL in units of cells per unit volume of blood, and the rate of loss of 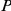 due to CTL is 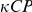. First, with this choice of units the parameter 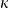 has units of 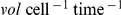 and is no longer interpretable as a rate of surveillance (see Text S1); rather, 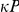 is the rate of loss of infected cells in blood per blood CTL. Second, using their estimate of 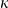 and measurements of 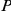, Wick *et al.* conclude that each CTL in blood kills approximately 0.7 infected cells per day. However, the loss of infected cells in blood is the also the result of killing of infected cells by CTL in spleen and other tissues; it is not clear how 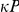 obtained from blood measurements relates to per-CTL killing rates in other anatomical locations.

## Discussion

Eliciting strong cellular immune responses has the potential to augment vaccine efficacy. To our knowledge, however, there are currently no estimates of how many CTL any given vaccine needs to generate, or even whether the necessary numbers are physiologically possible. Our approach provides first-order estimates of the required CTL densities that may inform the design of *in vivo* experiments or vaccine trials.

In a vaccinated individual, the E∶T ratio might be expected to be high at the beginning of an infection. In this case we have shown that handling times can be neglected and only the effective rate of CTL surveillance needs to be estimated to obtain the critical density. The effective surveillance rate combines (i) the rate at which CTL move between (survey) cells, (ii) the timecourse of expression of virus epitopes on infected cells, and (iii) the sensitivity of CTL to different levels of epitope expression. We illustrated this by estimating the critical CTL density required for the early control of SIV infection.

We have shown that considering virus life-histories is important for two reasons. First, using the simplest mass-action models of CTL killing with estimates of surveillance rates underestimates the number of CTL required to provide immunity. Second, intuition might have suggested that CTL are more effective against lytic viruses than budding viruses, as removing a cell infected with a lytic virus prevents all transmission from that cell. We show that the converse is true, after controlling for growth rate and infected cell lifetime. Thus knowing the visibility of infected cells to CTL, as well as the virus production schedule, is important for calculating critical CTL densities.

There other factors and potential refinements that need to be considered:

### Explicitly combining both models

We have discussed the issues of handling time and virus epitope dynamics separately, and have argued that only the latter needs to be considered at high E∶T ratios. When E∶T is low, a model incorporating both processes may be required. Text S2 describes such a model, and shows how both the models considered here can be derived from it.

### Spatial effects

Simulations suggest that the assumption of a mass-action killing rate may hold in some spatially structured environments [34]. However, as discussed for SIV, mass-action may not hold if infected cells are clustered, which may be likely particularly if infection takes place through cell-cell transmission; or if there is directed motion of CTL towards infected cells driven by chemotactic or inflammatory cues. Incorporating these factors requires the use of spatially explicit dynamical models such as those presented in [34]–[36]. Other effects may complicate the picture – for example, bystander killing of uninfected cells surrounding infected cells may provide a firebreak which limits further transmission [37], [38].

### Competition between CTL for infected cells

Reasonable estimates of the maximal densities of CTL achievable in a tissue might be the order of a few percent. Even at very high ratios of CTL to infected cells, then, if both populations are randomly distributed the probability of multiple CTL binding to a single infected cell is small. However, if infected cells exhibit clustering and/or if CTL migrate preferentially to infected cells, multiple CTL attachments to one cell may occur frequently [39], [40] and this effect needs to be considered in the calculation of numbers of effector cells not bound in conjugates.

### Multiple epitopes and CTL specificities

A CTL response typically comprises multiple clones with different functional quality and efficiencies of surveillance, and specific for different virus epitopes, each potentially with different timecourses of expression. Our analysis can be interpreted as either describing what is required of a single epitope-specific response, or the net effect of multiple CTL responses. In principle, the effect of multiple CTL responses can be calculated given a numerical and functional immunodominance hierarchy, and the timecourses of epitope expression [41]. Without going into this level of detail, however, it is very likely advantageous for a vaccine to induce a dominant CTL response to very early epitopes, and we show this reduces the estimate of the critical density.

### Stochastic effects

Very early in infection, when infected cell numbers are small, chains of transmission from cell to cell have a non-negligible probability of going extinct. The presence of CTL, even at a density insufficient to provide immunity once an infection reaches a deterministic phase of growth, may increase the probability of stopping virus growth very early in infection [42]. The use of high inocula in vaccine trials may mask this potential protective effect. Our calculation of the critical CTL density also assumes that the population of infected cells has reached a stable age distribution, which is a reasonable assumption following a small number of rounds of infected cell growth, a timescale that is likely comparable to that of activation of resident memory T cells. Explicit stochastic simulations may be required to study the dependence of the probability of early extinction on local CTL frequencies.

### Are sterilizing levels of CTL physiologically possible?

One concern with T cell based vaccines is that protection may only be possible with very high CTL densities, suggested by studies of CD8 T cell protection against the liver stage of Malaria infection [43], although in this system there may be significant spatial constraints on the surveillance of liver tissue by CTL. If this is the case, then according to the canonical model of a finite homeostatic capacity for T cell memory, the use of such vaccines may compromise the maintenance of existing memory T cell populations. However, it may be possible to generate very large numbers of CTL with prime-boost vaccination regimens without substantially ablating immunity to other pathogens [44].

In summary, our studies suggest that while there are many caveats with using models of CTL control of infected cell to understand infection dynamics, knowledge of life-history strategies may be important for refining our quantitative understanding of how CTL can contribute to the control of acute infections.

## Methods

All analyses were performed in 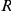 [45].

## Supporting Information

Text S1
**Mass-action killing and the interpretation of the rate constant** 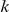 **.** The interpretation of the rate constant 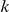 in equation (1) in the text when cell numbers are described as either dimensionless densities, as in the text, or as cells per unit volume.(PDF)

Text S2 **Modeling CTL killing with handling time and virus epitope dynamics.** A description of how the two models discussed here derive from a single model of CTL killing that incorporates both CTL-target handling time and the dynamics of virus epitope expression.
(PDF)

Text S3 **Derivation of the Lotka-Euler equation.** Derivation of equation (16) for the growth rate of a population of infected cells with a steady-state distribution of times since infection.
(PDF)
