# Supplementary material for: Virus Replication Strategies and the Critical CTL Numbers Required for the Control of Infection
Source: PLoS Comput Biol. 2011 Nov 17;7(11):e1002274. doi: 10.1371/journal.pcbi.1002274 (PMC3219614; doi:10.1371/journal.pcbi.1002274)
Supplement: Text S1 — Mass-action killing and the interpretation of the rate constant . The interpretation of the rate constant in equation (1) in the text when cell numbers are described as either dimensionless densities, as in the text, or as cells per unit volume. (PDF) [file pcbi.1002274.s001.pdf]

Text S1: Mass-action killing and the interpretation of the rate constant k

In a region of tissue containing N cells, suppose a proportion f_C are CTL and a proportion f_I are infected cells, we assume f_I is small and sufficiently small that susceptible cells are abundant, and the infected cell population is growing at rate r. These proportions are the densities referred to in the text. Then a mass-action model of killing of these infected cells by CTL is

$$\frac{\mathrm{d}(f_I N)}{\mathrm{d}t} = r f_I N - K(f_C N)(f_I N). \tag{1}$$

This assumes that CTL and infected cells are well mixed and moving randomly. The interpretation of the last term is as follows; $K(f_C N)$ is the probability per unit time (the rate parameter defining the underlying Poisson process) of any particular cell, infected or not, being encountered by any CTL; K is the probability per unit time of a given CTL encountering this cell; and KN is the probability per unit time of a given CTL encountering any cell at all; this is the rate of surveillance by a single CTL. The expected number of cells encountered in unit time by one CTL is then $1/(KN)$.

Equation (1) is then

$$\frac{\mathrm{d}f_I}{\mathrm{d}t} = r f_I - k f_C\, f_I. \tag{2}$$

where $k = KN$. This parameter k is the CTL surveillance rate (total number of cells surveyed per unit time per CTL) estimated in [1] and [2].

When C and I are measured as cells per unit volume, the interpretation of the constant of proportionality k in the loss rate kCI is different; k now contains dimensions of volume and so kI is the rate that a single CTL encounters infected cells present at a density I per unit volume. If the total density of surveyable cells in a tissue is S, kS can be interpreted as the rate of surveillance.

References

1. Regoes RR, Barber DL, Ahmed R, Antia R (2007) Estimation of the rate of killing by cytotoxic T lymphocytes in vivo. Proc Natl Acad Sci U S A 104: 1599-603.
2. Yates A, Graw F, Barber DL, Ahmed R, Regoes RR, et al. (2007) Revisiting estimates of CTL killing rates in vivo. PLoS One 2: e1301.
