# Supplementary material for: Virus Replication Strategies and the Critical CTL Numbers Required for the Control of Infection
Source: PLoS Comput Biol. 2011 Nov 17;7(11):e1002274. doi: 10.1371/journal.pcbi.1002274 (PMC3219614; doi:10.1371/journal.pcbi.1002274)
Supplement: Text S2 — Modeling CTL killing with handling time and virus epitope dynamics. A description of how the two models discussed here derive from a single model of CTL killing that incorporates both CTL-target handling time and the dynamics of virus epitope expression. (PDF) [file pcbi.1002274.s002.pdf]

Text S2: Modeling CTL killing with handling time and virus epitope dynamics

The models we study in this paper are instances of a more general model of the interactions between CTL and infected cells structured with respect to their age since infection. The model is illustrated in the figure and described below.

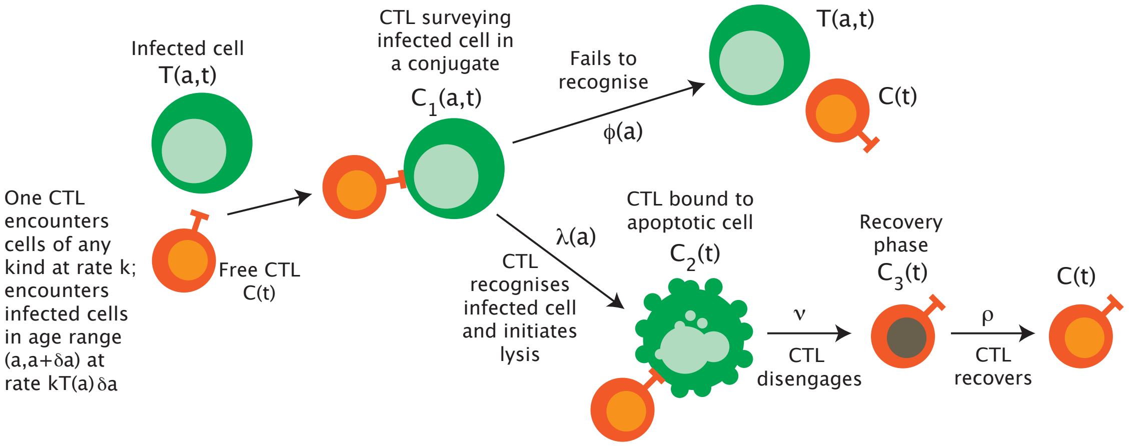

Figure 1. A model of CTL killing incorporating (i) the efficiency of detection of infected cells as a function of age since infection, and (ii) the time taken for CTL to lyse their targets.

$$\frac{\partial T}{\partial t} + \frac{\partial T}{\partial a} = -\left(kC(t) + \mu(a)\right) T(a,t) + \phi(a) C_1(a,t) \quad (1)$$

$$T(a=0,t) = \epsilon \int_0^\infty m(a) T(a,t)\, da \quad (2)$$

$$\frac{\mathrm{d}C}{\mathrm{d}t} = \rho C_3(t) + \int_0^\infty \left\{ (\mu(a) + \phi(a)) C_1(a,t) - kC(t)T(a,t) \right\} da \quad (3)$$

$$\frac{\partial C_1}{\partial t} + \frac{\partial C_1}{\partial a} = kC(t)T(a,t) - (\mu(a) + \phi(a) + \lambda(a)) C_1(a,t) \quad (4)$$

$$\frac{\mathrm{d}C_2}{\mathrm{d}t} = \int_0^\infty \lambda(a) C_1(a,t)\, da - \nu C_2(t) \quad (5)$$

$$\frac{\mathrm{d}C_3}{\mathrm{d}t} = \nu C_2(t) - \rho C_3(t) \quad (6)$$

The populations T, C, C_1, C_2 and C_3 are defined as follows. $T(a,t)$ is the population density of infected cells of age a in the tissue, such that $\int_a^{a+\delta a} T(a,t)\, da$ is the density of cells at time t with an age since infection in the range $(a, a+\delta a)$; the total density of infected cells is $\int_0^\infty T(a,t)\, da \in [0,1]$. $C(t) \in [0,1]$ is the density of free CTL; $C_1(a,t)$ is the density of CTL that are conjugated to live cells

of infection age a and surveying them, such that $\int C_1(a,t)\,da \in [0,1]$; $C_2(t) \in [0,1]$ is the density of CTL conjugated to infected cells during lysis; and $C_3(t) \in [0,1]$ is the density of CTL recently disengaged from a lytic event but unable to kill.

The parameters are defined as follows:

- k is the rate that a CTL moves between cells, such that $1/k$ is the expected time to a CTL's next encounter with any cell, and $(k \int T(a,t)da)^{-1}$ is the expected time to a given CTL's next encounter with an infected cell of any age.
- $\mu(a)$ is the age-dependent rate of loss of cells due to non-CTL mediated mechanisms such as natural mortality and viral cytopathicity.
- $m(a)$ is the rate of production of virions by an infected cell at age a after infection.
- ϵ is the mean number of infected cells arising from a single virion.
- $\phi(a)$ is the rate at which a CTL conjugated with an infected cell of age a disengages without lysis.
- $\lambda(a)$ is the rate at which, following an encounter, a CTL successfully identifies an infected cell of age a and begins the process of lysis.
- ν is the rate at which a CTL disengages from an apoptotic cell; so $1/\nu$ is the mean time a CTL spends attached to a cell following the initiation of lysis.
- ρ is the rate at which a disengaged CTL recovers and again becomes able to detect infected cells.

Implicit in the equations above is a quasi-steady state assumption for the dynamics of free virus, and the assumption that susceptible cells are in abundance. We assume that free virus has a very short half-life in the extracellular space and so the rate of infection is directly proportional to the total rate of virus production.

The first model in the text (eqns. 4-6) derives from this model as follows. All quantities are independent of the age since infection, a; CTL recognise infected cells immediately following encounter ($\phi = 0$ and $\lambda = \infty$); there is no natural mortality of infected cells ($\mu = 0$) and no spread of infection from cell to cell ($m = \epsilon = 0$); CTL-infected cell conjugates break up at rate ν, and CTL recovery is assumed to be included in this process ($\rho = \infty$).

The second model (eqns. 13 and 14) derives from it as follows. Following a CTL's encounter with an infected cell of age a, it is recognised as infected with probability $p(a) \equiv \lambda(a)/(\lambda(a) + \phi(a))$, killed instantly ($\lambda(a) \to \infty$ and $\phi(a) \to \infty$ such that $p(a)$ is invariant; and $\nu = \infty$) and the CTL recovers instantly ($\rho = \infty$). This yields the age-specific rate of surveillance $k(a) = p(a)k$.
