# Supplementary material for: Virus Replication Strategies and the Critical CTL Numbers Required for the Control of Infection
Source: PLoS Comput Biol. 2011 Nov 17;7(11):e1002274. doi: 10.1371/journal.pcbi.1002274 (PMC3219614; doi:10.1371/journal.pcbi.1002274)
Supplement: Text S3 — Derivation of the Lotka-Euler equation. Derivation of equation (16) for the growth rate of a population of infected cells with a steady-state distribution of times since infection. (PDF) [file pcbi.1002274.s003.pdf]

Text S3: Derivation of the Lotka-Euler equation

The Lotka-Euler equation (Equation 16) can be derived as follows. The total number of infected cells in age class a at time t is the cells that were infected a time a ago and survived to age a;

$$I(a,t) = \ell(a)I(0,t-a) = \epsilon\ell(a)\int_{s=0}^{\infty} I(s,t-a)m(s)ds$$

We are assuming steady state exponential growth of infected cells in all age-classes, $I(a,t) = e^{rt}I(a,0)$, so

$$\begin{aligned} I(a,t) &= \epsilon\,\ell(a)\int_{s=0}^{\infty} I(s,t)e^{-ra}m(s)ds \\ &= \epsilon\,\ell(a)e^{-ra}\int_{s=0}^{\infty} I(s,t)m(s)ds \end{aligned}$$

The total rate of virus production at time t is then

$$\int_a I(a,t)m(a)da = \epsilon\int_a m(a)\ell(a)e^{-ra}da\int_s I(s,t)m(s)ds$$

which gives Equation 16 in the main text.
